# Biochemical and Inflammatory Profiles in Paediatric Obesity, Undernutrition and Normal Weight: A Single-Centre Retrospective Cohort Study

**DOI:** 10.3390/children13080975

**Published:** 2026-07-23

**Authors:** Taner Adıgüzel, Erdin Kalyoncuoğlu, Gülsüm Kaya

**Affiliations:** 1 Department of Pediatrics, Faculty of Medicine, Yalova University, 77200 Yalova, Türkiye; erdin.kalyoncuoglu@saglik.gov.tr; 2 Department of Medical Microbiology, Faculty of Medicine, Yalova University, 77200 Yalova, Türkiye; gulsum.kaya@yalova.edu.tr

**Keywords:** pediatric obesity, undernutrition, body mass index, alanine aminotransferase, AST/ALT ratio, 25-hydroxyvitamin D, CBC-derived inflammatory indices, insulin resistance

## Abstract

**Highlights:**

**What are the main findings?**

• Childhood obesity was associated with a distinct pattern of liver enzyme, metabolic and inflammatory laboratory abnormalities compared with undernutrition and normal weight.
• The aspartate aminotransferase-to-alanine aminotransferase ratio showed the strongest ability to distinguish children with obesity among the evaluated routine laboratory markers.
• Undernutrition demonstrated a different biochemical and micronutrient profile rather than simply the opposite pattern of obesity.

**What are the implications of the main findings?**

• Routine laboratory tests may help identify children with obesity who require further metabolic and liver evaluation.
• The aspartate aminotransferase-to-alanine aminotransferase ratio and systemic inflammation response index deserve prospective validation as practical screening biomarkers.
• Larger prospective studies with age-balanced cohorts and liver imaging are needed before these findings can be applied in clinical practice.

**Abstract:**

**Background**: We compared biochemical, micronutrient-related and complete blood count (CBC)-derived inflammatory profiles among children with obesity, undernutrition and normal weight, and explored the discriminatory performance of selected routine laboratory markers for the obesity phenotype. **Methods**: In this single-centre retrospective comparative study, medical records of children aged 2–17 years evaluated between January 2023 and December 2025 were reviewed. Participants were classified as obese, undernourished or normal weight using World Health Organization (WHO) body mass index-for-age (BMI-for-age) references. Liver enzymes, lipid parameters, iron-related indices, micronutrient levels, zinc, insulin and CBC-derived inflammatory indices were compared across groups using non-parametric omnibus tests. Exploratory receiver operating characteristic (ROC) analyses were performed for selected markers. **Results**: A total of 690 children were included: 100 with obesity, 90 with undernutrition and 500 with normal weight. The obesity group was significantly older than the normal-weight group (median 11.0 vs. 7.5 years; *p* < 0.001). Obesity was characterised by higher alanine aminotransferase (ALT), insulin, triglycerides, neutrophil-to-lymphocyte ratio (NLR), systemic immune-inflammation index (SII) and systemic inflammation response index (SIRI), together with lower high-density lipoprotein (HDL) cholesterol, 25-hydroxyvitamin D [25(OH)D] and aspartate aminotransferase-to-alanine aminotransferase (AST/ALT) ratio (all *p* < 0.05). In exploratory, age-unadjusted ROC analyses, the AST/ALT ratio yielded the highest AUC among the evaluated biomarkers (AUC 0.859; 95% CI 0.823–0.895; optimal cut-off ≤ 1.52; sensitivity 77.0%; specificity 77.6%), followed by SIRI (AUC 0.751; 95% CI 0.700–0.802). **Conclusions**: Pediatric obesity was associated with a distinct liver enzyme-metabolic-inflammatory laboratory profile, whereas undernutrition exhibited a different micronutrient-biochemical pattern. Because the study was retrospective and age-unadjusted, these findings should be interpreted as exploratory and hypothesis-generating. Prospective studies with age-balanced sampling, pubertal staging and liver imaging are required before clinical implementation.

## 1. Introduction

Childhood obesity is a chronic non-communicable disease associated with metabolic dysregulation, organ-specific complications, and long-term cardiometabolic risk beginning early in life [1]. At the opposite end of the nutritional spectrum, undernutrition remains a major paediatric public health problem, adversely affecting growth, immune function, neurodevelopment, and susceptibility to infection [2]. These conditions may coexist within the same population, representing the double burden of malnutrition [3].

Despite their contrasting phenotypes, both obesity and undernutrition are associated with metabolic and inflammatory alterations. Paediatric obesity is characterized by chronic low-grade inflammation, dyslipidaemia, insulin resistance, and liver enzyme abnormalities [4,5,6], whereas undernutrition is associated with catabolic adaptation, altered hepatic metabolism, and micronutrient deficiencies [7,8]. Comparing these nutritional phenotypes may improve our understanding of their distinct biochemical profiles.

From a hepatic perspective, obesity is strongly associated with metabolic dysfunction-associated steatotic liver disease (MASLD) [9,10,11,12]. Because liver imaging was not routinely available in this retrospective cohort, we evaluated serum ALT, AST, and the AST/ALT ratio as readily available biochemical indicators of hepatic involvement [10,11,12,13].

Lower 25(OH)D concentrations are frequently reported in children with obesity [4,14], while findings regarding vitamin B12, folate, and trace elements remain inconsistent [15]. In addition, complete blood count (CBC)-derived inflammatory indices, including the neutrophil-to-lymphocyte ratio (NLR), platelet-to-lymphocyte ratio (PLR), systemic immune-inflammation index (SII), and systemic inflammation response index (SIRI), have emerged as inexpensive markers of obesity-related inflammation [16,17,18].

Interpretation of these laboratory markers in children is further complicated by normal developmental changes. Liver enzyme activity, lipid metabolism, insulin sensitivity, and CBC-derived inflammatory indices may vary with age and pubertal maturation because of physiological hormonal and metabolic changes during growth. Consequently, age and pubertal status should be considered important potential confounding factors when comparing metabolic and inflammatory biomarkers across paediatric populations.

To our knowledge, few studies have simultaneously compared liver biochemical markers, CBC-derived inflammatory indices, and micronutrient-related parameters across obesity, normal-weight, and undernutrition phenotypes in children. Identifying routinely available laboratory markers that distinguish these nutritional phenotypes may assist clinicians in the initial metabolic assessment of pediatric patients and help prioritize those who may benefit from further evaluation.

We hypothesised that children with obesity would demonstrate a distinct hepatic-inflammatory laboratory profile characterized by a lower AST/ALT ratio and increased CBC-derived inflammatory indices compared with normal-weight and undernourished children. Accordingly, the primary objective of this study was to compare these biomarkers across the three nutritional phenotypes. The secondary objective was to explore the discriminatory performance of selected routine laboratory markers for identifying the obesity phenotype using exploratory, age-unadjusted ROC analyses.

## 2. Materials and Methods

### 2.1. Study Design and Setting

This was a single-centre retrospective observational comparative study based on the medical records of paediatric patients evaluated at a tertiary training and research hospital between January 2023 and December 2025. Reporting was structured in accordance with the Strengthening the Reporting of Observational Studies in Epidemiology (STROBE) statement [19].

### 2.2. Participants

Children aged 2–17 years were eligible if anthropometric measurements and the principal laboratory parameters of interest were available from the same clinical evaluation period. The final group sizes reflected all eligible records identified during the study period; no prespecified matching or equal-group allocation was applied.

The normal-weight group consisted of children with a body mass index (BMI) between the 5th and 85th percentiles who attended the pediatric outpatient clinic during the study period and underwent laboratory evaluation for clinical indications unrelated to obesity or malnutrition. These participants were identified retrospectively from hospital records and served as a hospital-based comparison cohort rather than a community-based healthy control population. The same inclusion and exclusion criteria were applied across all study groups. Children with acute or chronic inflammatory diseases, endocrine disorders, malignancy, autoimmune diseases, or other conditions known to affect inflammatory or biochemical parameters were excluded.

Because of the retrospective study design, some secondary laboratory variables (25-hydroxyvitamin D, vitamin B12, and ferritin) were unavailable for all participants, as these tests were requested according to clinical indications rather than as part of a standardized study protocol. No imputation of missing values was performed. Analyses involving these variables were conducted using complete-case analysis, including only participants with available data. The primary study variables (AST, ALT, AST/ALT ratio, lipid profile, and CBC-derived inflammatory indices) were available for all included participants.

Because this was a retrospective study based on routinely collected clinical data, pubertal staging (Tanner stage) was not consistently documented and therefore could not be incorporated into the analyses. Together with the significant age imbalance between groups, this represents an inherent limitation of the study design that should be considered when interpreting between-group differences.

### 2.3. Exclusion Criteria

To minimise distortion of CBC-derived inflammatory indices by intercurrent illness, records were excluded if there was documented acute infection at the time of blood sampling, overt inflammatory disease or an elevated C-reactive protein value. Where clearly documented in the chart, chronic liver disease, haematological or oncological diagnosis and ongoing systemic corticosteroid or immunosuppressive therapy were also considered exclusionary. Because comorbidity and medication data were not uniformly structured across all retrospective records, residual confounding from undocumented conditions cannot be fully excluded.

### 2.4. Anthropometric Classification

Children aged 2–4 years were classified using WHO Child Growth Standards [20] and those aged 5–17 years using WHO BMI-for-age reference charts [21]. Obesity was operationally defined as BMI-for-age > +2 standard deviations (SD) and undernutrition as BMI-for-age < −2 SD. Children within the reference range without overweight or obesity constituted the normal-weight group. The term undernutrition was used deliberately to denote low BMI-for-age status and to distinguish it from the broader concept of malnutrition, which encompasses obesity [3].

### 2.5. Laboratory Measurements

Variables obtained during the same evaluation period included: glucose, creatinine, total protein, albumin, total cholesterol, triglycerides, HDL cholesterol, low-density lipoprotein (LDL) cholesterol, AST, ALT, iron, total iron-binding capacity (TIBC), vitamin B12, 25(OH)D, folate, insulin, haemoglobin A1c (HbA1c), zinc and CBC parameters. Analyses were performed in the hospital’s central biochemistry and haematology laboratories using standard automated methods. Fasting status could not be verified for all retrospective lipid and insulin measurements; accordingly, these values were interpreted as real-world biochemical markers rather than uniformly fasting values. For contextual interpretation, ALT findings were discussed with reference to sex-specific paediatric thresholds from the SAFETY study [22] and 25(OH)D values were evaluated in line with current international recommendations [23].

### 2.6. CBC-Derived Inflammatory Indices

The NLR, PLR, SII and SIRI were calculated using standard formulae: NLR = neutrophil count ÷ lymphocyte count; PLR = platelet count ÷ lymphocyte count; SII = (platelet count × neutrophil count) ÷ lymphocyte count; SIRI = (monocyte count × neutrophil count) ÷ lymphocyte count.

### 2.7. Ethics Approval and Consent to Participate

This study was approved by the Yalova University Non-Interventional Health Sciences Ethics Committee (Protocol No. 2026/64; Approval Date: 1 April 2026). All procedures involving human participants were conducted in accordance with the ethical standards of the Yalova University Non-Interventional Health Sciences Ethics Committee and with the Declaration of Helsinki (1964) and its later amendments. Owing to the retrospective study design and the use of anonymized medical records, the requirement for informed consent was waived by the Ethics Committee.

### 2.8. Statistical Analysis

Data were analysed using IBM SPSS Statistics for Windows, version 22.0 (IBM Corp., Armonk, NY, USA). Continuous variables are presented as mean ± standard deviation (SD) or median [interquartile range (IQR)] according to distribution, and categorical variables as number and percentage. Normality was assessed using the Kolmogorov–Smirnov test. Between-group omnibus comparisons were performed using one-way analysis of variance (ANOVA) for normally distributed variables and the Kruskal–Wallis test for non-normally distributed variables; categorical variables were compared using the chi-square or Fisher exact test, as appropriate. Extreme laboratory values were verified against the source dataset for data-entry accuracy before final analysis. When the overall Kruskal–Wallis test was significant, pairwise post hoc comparisons were performed using the Mann–Whitney U test with Bonferroni correction. Because three pairwise comparisons were conducted, statistical significance for post-hoc analyses was defined as *p* < 0.0167.

To address the potential confounding effect of age, a multivariable binary logistic regression analysis was performed using the enter method. Obesity status (obese vs. non-obese) was entered as the dependent variable, while age, sex, AST, AST/ALT ratio, triglycerides, HDL cholesterol, and SIRI were entered as independent variables. Adjusted odds ratios (ORs) with 95% confidence intervals (95% CIs) were calculated, and a two-sided *p* value < 0.05 was considered statistically significant.

Receiver operating characteristic (ROC) curve analyses were performed to explore the discriminatory ability of selected laboratory markers for the obesity phenotype. Because of the significant age imbalance between the study groups, these analyses were considered exploratory and were not adjusted for age. Accordingly, the reported AUC values, optimal cut-off points, sensitivity, and specificity estimates should not be interpreted as reflecting age-independent diagnostic performance, but rather as hypothesis-generating findings requiring prospective validation. Given the marked age imbalance between the study groups and the absence of pubertal staging (Tanner stage) data, the independent effects of age, pubertal maturation, and nutritional status could not be fully disentangled. Consequently, comprehensive adjustment for these developmental factors was not feasible. The study was therefore designed and interpreted as a descriptive, comparative, and hypothesis-generating investigation rather than one intended to establish causal or age-independent associations.

## 3. Results

Participants and baseline characteristics (Table 1). A total of 690 children were included: 100 with obesity, 90 with undernutrition and 500 with normal weight. Age differed significantly across groups (*p* < 0.001): the obesity group (median 11.0 [8.5–15.0] years) was markedly older than the normal-weight group (7.5 [5.0–11.5] years), with the undernutrition group intermediate (10.0 [6.5–12.1] years). Sex distribution was similar across groups (*p* = 0.236). This age imbalance is an important potential confound for all metabolic and inflammatory comparisons and is addressed in the Limitations section.

Liver enzyme and metabolic findings (Table 2). The obesity group had the highest median ALT (18.3 [14.0–25.0] U/L vs. 13.0 [10.9–17.0] in undernutrition vs. 12.0 [10.0–16.0] in normal weight; *p* < 0.001) and the lowest median AST/ALT ratio (1.2 [0.8–1.5] vs. 1.9 [1.5–2.2] vs. 2.0 [1.5–2.4]; *p* < 0.001). Conversely, AST was lowest in the obesity group (22.0 [19.0–26.7] U/L), consistent with disproportionate ALT elevation. Insulin was markedly higher in obesity (19.7 [15.7–27.6] µIU/mL) than in undernutrition (5.8 [4.5–10.2]) and normal weight (6.9 [4.2–13.8]; *p* < 0.001). HbA1c did not differ significantly across groups (*p* = 0.316), consistent with compensatory hyperinsulinaemia without overt glycaemic failure. Total protein and albumin values also differed across groups (*p* < 0.001 and *p* = 0.010, respectively), with the highest medians in the obesity group.

Lipid and micronutrient profile (Table 2). Triglycerides were highest in obesity (87.7 [68.1–127.5] mg/dL; *p* < 0.001) and HDL cholesterol was lowest (47.5 [42.0–56.5] mg/dL; *p* = 0.004), whereas total and LDL cholesterol did not differ significantly across groups (*p* = 0.478 and *p* = 0.183, respectively). Obesity was associated with the lowest median 25(OH)D (12.8 [9.4–18.8] ng/mL vs. 18.3 [13.4–25.3] in undernutrition and 18.4 [13.0–24.2] in normal weight; *p* < 0.001) and the lowest vitamin B12 (367.1 ± 154.6 pg/mL; *p* = 0.006). Folate was highest in undernutrition (*p* = 0.017). Iron was lowest in obesity (*p* = 0.009), whereas TIBC was paradoxically highest in the obesity group (359.4 [302.4–386.4] µg/dL vs. approximately 322 µg/dL in the other groups; *p* < 0.001). Zinc differed across groups (*p* = 0.016), with the highest median values in undernutrition.

CBC-derived inflammatory indices (Table 3). Significant overall differences were observed among the three groups for all four inflammatory indices. Post hoc Mann–Whitney U analyses showed that NLR was significantly higher in the obesity group than in both the undernutrition (*p* = 0.003) and normal-weight groups (*p* < 0.001), whereas no significant difference was observed between the undernutrition and normal-weight groups (*p* = 0.647). SIRI also differed significantly between all three groups (obesity vs. undernutrition, *p* = 0.002; obesity vs. normal weight, *p* < 0.001; undernutrition vs. normal weight, *p* < 0.001). PLR and SII showed significant overall between-group differences (*p* = 0.005 and *p* < 0.001, respectively); however, no formal pairwise post hoc comparisons were performed for these indices.

Pairwise post hoc comparisons using the Mann–Whitney U test with Bonferroni correction showed that the obesity group had significantly higher AST, ALT, triglyceride, NLR, PLR, SII, and SIRI levels, as well as a significantly lower HDL cholesterol level and AST/ALT ratio, compared with the normal-weight group (all adjusted *p* < 0.0167). The obesity and undernutrition groups differed significantly only in HDL cholesterol and the AST/ALT ratio (both adjusted *p* < 0.0167), whereas no significant differences were observed for AST, ALT, triglycerides, NLR, PLR, SII, or SIRI. Between the undernutrition and normal-weight groups, significant differences were observed only for triglycerides and SIRI (both adjusted *p* < 0.0167), while AST, ALT, AST/ALT ratio, HDL cholesterol, NLR, PLR, and SII did not differ significantly (Table 4).

To address the potential confounding effect of age, an additional multivariable logistic regression analysis including age and sex as covariates was performed (Table 5). After adjustment, a lower AST/ALT ratio (adjusted OR = 0.043, 95% CI 0.021–0.089; *p* < 0.001), higher triglyceride levels (adjusted OR = 1.007, 95% CI 1.001–1.013; *p* = 0.025), and higher SIRI values (adjusted OR = 5.225, 95% CI 2.920–9.351; *p* < 0.001) remained independently associated with the obesity phenotype. In contrast, AST, HDL cholesterol, age, and sex were not independently associated with obesity after adjustment (*p* > 0.05).

**Figure 1:**
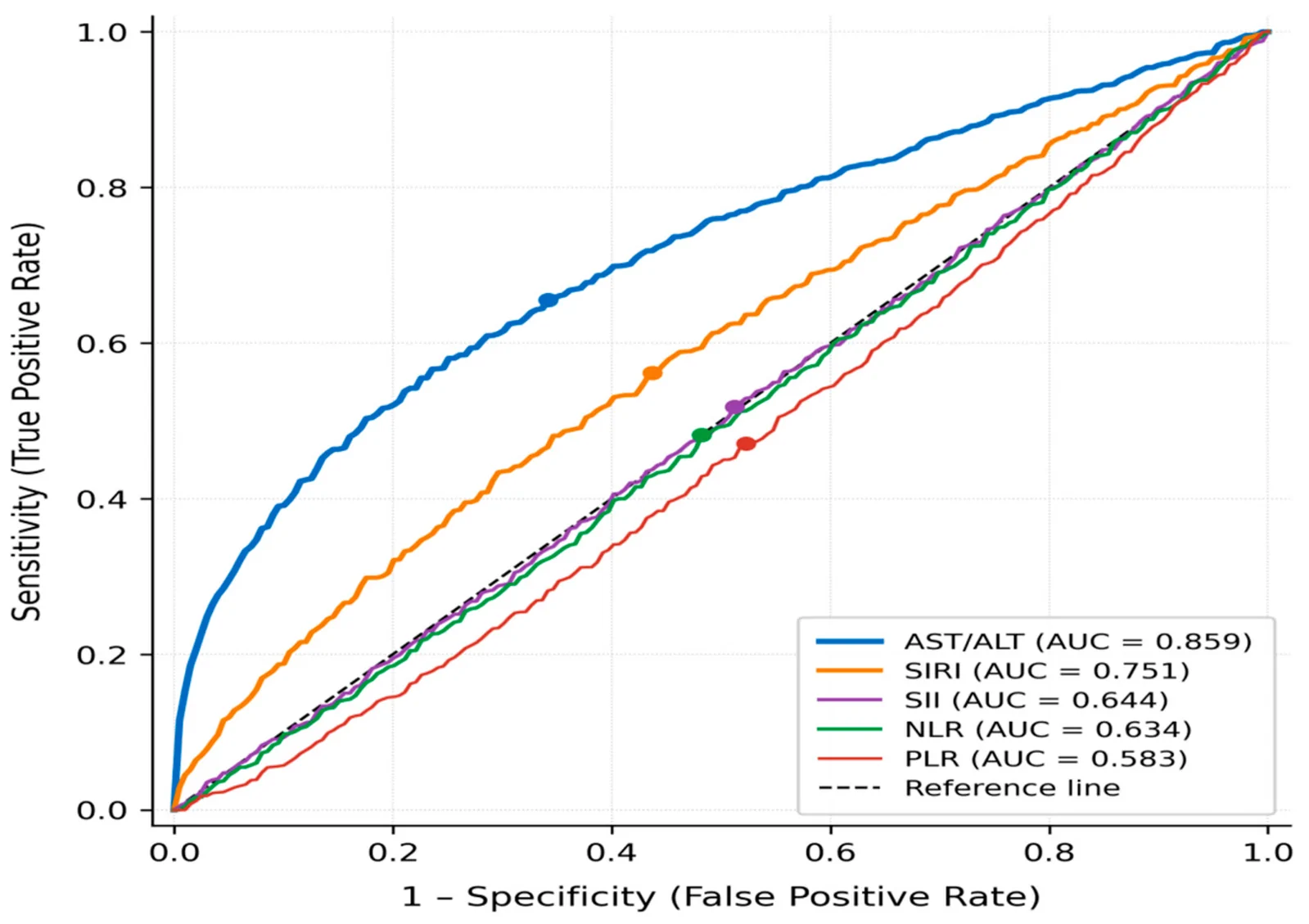
Receiver operating characteristic (ROC) curves for the age-unadjusted exploratory discriminatory performance of selected markers for the paediatric obesity phenotype. Operating points (●) indicate optimal cut-off values identified by the Youden index. For the AST/ALT ratio, lower values are associated with the obesity phenotype (cut-off ≤ 1.52); the curve was therefore plotted with decreasing ratio as the positive test direction. Analyses were performed without age adjustment; AUC estimates reflect age-confounded discrimination. Curves are ordered in the legend by descending AUC. AST/ALT, aspartate aminotransferase-to-alanine aminotransferase ratio; AUC, area under the curve; NLR, neutrophil-to-lymphocyte ratio; PLR, platelet-to-lymphocyte ratio; ROC, receiver operating characteristic; SII, systemic immune-inflammation index; SIRI, systemic inflammation response index.

Age-unadjusted exploratory ROC analysis (Table 6; Figure 1). The AST/ALT ratio yielded the highest AUC among the evaluated biomarkers (AUC 0.859; 95% CI 0.823–0.895; cut-off ≤1.52; sensitivity 77.0%; specificity 77.6%), followed by SIRI (AUC 0.751; 95% CI 0.700–0.802; cut-off > 0.70; sensitivity 69.0%; specificity 70.8%). SII (AUC 0.644) and NLR (AUC 0.634) showed modest but statistically significant discriminatory performance, whereas PLR had the lowest AUC (0.583).

## 4. Discussion

In this hospital-based paediatric cohort of 690 children, obesity was associated with a distinct laboratory pattern comprising elevated ALT, a reduced AST/ALT ratio, hyperinsulinaemia, hypertriglyceridaemia, reduced HDL cholesterol, lower 25(OH)D and elevated CBC-derived inflammatory indices. Undernutrition, by contrast, was characterised by higher AST and differences in zinc, folate and iron-related indices. Taken together, these findings suggest that opposite BMI-for-age phenotypes are accompanied by partly distinct—rather than simply inverse—laboratory signatures within the same clinical setting, consistent with the concept of the double burden of malnutrition. Because the obesity group was older than the normal-weight group, part of the observed metabolic and inflammatory separation may reflect age- or puberty-related biological differences rather than BMI phenotype alone.

Liver biochemistry and the AST/ALT ratio. The lower AST/ALT ratio observed in obesity is compatible with an ALT-predominant biochemical pattern described in paediatric obesity and MASLD-related literature, which may reflect obesity-related hepatic metabolic stress rather than diagnostic evidence of hepatic steatosis [10,11,12,13]. In the present exploratory, age-unadjusted analysis, an AST/ALT ratio ≤ 1.52 was identified as the optimal cut-off for discriminating the obesity phenotype within this study population. However, because the analyses were not adjusted for age, pubertal staging was unavailable, and liver imaging was not performed, this cut-off should be considered hypothesis-generating rather than clinically applicable. External validation in independent, age-balanced cohorts with standardized hepatic assessment is required before any diagnostic or screening role can be considered.

Although the AST/ALT ratio yielded the highest AUC among the evaluated biomarkers in the exploratory ROC analysis, these findings should be interpreted cautiously and should not be regarded as evidence of clinical diagnostic accuracy. Because the ROC analyses were exploratory and were not adjusted for age, and the obesity group was significantly older than the comparison groups, part of the observed discriminatory performance may reflect age-related physiological differences rather than obesity itself. Furthermore, the retrospective single-centre design precludes conclusions regarding clinical diagnostic utility. Importantly, however, the association between a lower AST/ALT ratio and the obesity phenotype remained statistically significant after adjustment for age and sex in the multivariable logistic regression analysis, suggesting that the observed association is not solely explained by age imbalance. Nevertheless, prospective validation in independent, age-balanced cohorts is required before the AST/ALT ratio can be considered for clinical application.

Insulin resistance and lipid profile. Markedly elevated insulin without corresponding HbA1c elevation is consistent with compensatory hyperinsulinaemia reflecting early insulin resistance before overt β-cell failure [6]. The co-occurrence of hypertriglyceridaemia and low HDL cholesterol further supports an early atherogenic, insulin-resistant metabolic phenotype in this obesity cohort [5,6]. The absence of significant total and LDL cholesterol differences across groups may reflect the relatively early metabolic stage of this cohort or dilution by the large, younger normal-weight group.

Vitamin D and micronutrients. Lower 25(OH)D in obesity is concordant with published paediatric meta-analyses and is attributed to adipose sequestration, reduced outdoor activity and suboptimal dietary intake [4,14]. Lower vitamin B12 in obesity may reflect dilutional effects, altered dietary patterns or reduced absorption. The paradoxically elevated TIBC observed in the obesity group should be interpreted cautiously. Although co-existing iron deficiency or functional iron deficiency may represent possible explanations, these mechanisms remain speculative because ferritin and other comprehensive markers of iron metabolism were not available for all participants. Therefore, the present data do not allow conclusions regarding the underlying mechanisms of the observed TIBC elevation, and further studies incorporating a complete assessment of iron metabolism are warranted.

Undernutrition findings. The undernutrition group did not display a simple biochemical reversal of the obesity pattern. Higher AST and differences in zinc, folate and iron-related variables suggest a distinct hepatic and micronutrient adaptation profile, potentially reflecting catabolic substrate utilisation, reduced lean body mass, supplementation history or referral-related selection factors [7,8]. Clinically, these findings reinforce the view that undernutrition and obesity are not biochemical mirror images and that both phenotypes warrant condition-specific laboratory evaluation rather than a single generic panel.

The observed elevations in CBC-derived inflammatory indices in children with obesity are consistent with the concept of obesity-associated low-grade systemic inflammation described in previous studies [16,17,18]. SIRI showed the most pronounced proportional elevation and ranked second in ROC performance, suggesting potential utility as a composite inflammatory signal. These indices are derived from the standard CBC and are universally accessible without additional cost; however, they are non-specific and require clinical contextualisation. Their elevation is also liable to age confounding in this dataset, as discussed below.

Strengths of this study include the simultaneous three-group single-cohort design, the broad panel of routine biochemical, micronutrient and inflammatory markers assessed concurrently, transparent reporting in line with STROBE, and the acknowledgement of methodological constraints throughout the manuscript.

This study adds to the existing literature by simultaneously evaluating liver biochemical markers, CBC-derived inflammatory indices, and micronutrient-related parameters across three pediatric nutritional phenotypes within the same cohort. While previous studies have primarily compared children with obesity and normal weight, the inclusion of an undernutrition group allowed a broader characterization of nutritional extremes and demonstrated that the biochemical profile associated with obesity is not simply the inverse of undernutrition. These findings provide a more comprehensive understanding of the metabolic and inflammatory alterations associated with different nutritional states in children.

From a clinical perspective, the present findings do not support the use of the AST/ALT ratio or inflammatory indices as stand-alone diagnostic tools. Rather, they suggest that routinely available laboratory parameters obtained during standard clinical evaluation may provide complementary information regarding the metabolic and inflammatory profile of children with obesity. In particular, the persistence of the association between a lower AST/ALT ratio and obesity after adjustment for age and sex suggests that this biomarker may contribute to risk stratification alongside established clinical assessment, although prospective validation is required before incorporation into routine practice.

### Limitations

The principal limitation is the significant age imbalance between groups: the obesity cohort was approximately 3.5 years older than the normal-weight group. Insulin sensitivity, lipid profiles, CBC-derived inflammatory indices and liver biochemistry all change substantially across childhood and at puberty. Without pubertal staging (Tanner data were unavailable) or multivariable age-adjusted modelling, the observed group differences cannot be fully attributed to nutritional phenotype alone. All ROC analyses are therefore age-unadjusted and the AUC estimates reflect age-confounded discrimination. Additional limitations include: the retrospective single-centre design; the hospital-based rather than community-derived normal-weight comparison group, which may not represent the general paediatric population; unverified fasting status for lipid and insulin measurements; the absence of liver imaging to confirm hepatic steatosis; and the sample size imbalance (n = 500 normal weight vs. n = 100 obesity vs. n = 90 undernutrition), which may have inflated statistical power for differences involving the large normal-weight group. Prospective studies with age-stratified or age-matched sampling, Tanner staging, standardised fasting conditions, adiposity phenotyping (waist-to-height ratio or body composition analysis) and liver imaging are required before causal inferences or clinical implementation can be considered. In addition, although participants were classified using age- and sex-specific BMI percentile categories, BMI z-scores or percentage of the 95th BMI percentile were not available for analysis. These measures may have provided a more standardized assessment of adiposity across the broad pediatric age range and should be considered in future studies.

## 5. Conclusions

In this retrospective single-centre paediatric cohort, obesity was associated with a combined liver enzyme-metabolic-inflammatory laboratory pattern—characterised by elevated ALT, a reduced AST/ALT ratio, hyperinsulinaemia, hypertriglyceridaemia, low HDL cholesterol, lower 25(OH)D and elevated CBC-derived inflammatory indices—whereas undernutrition showed a distinct micronutrient-biochemical profile. Among the evaluated biomarkers, the AST/ALT ratio yielded the highest AUC in exploratory, age-unadjusted ROC analyses (AUC 0.859), followed by SIRI (AUC 0.751). However, these findings should be considered exploratory and hypothesis-generating, and require validation in prospective studies with age-balanced cohorts before clinical application. Because the study was retrospective, substantially age-unadjusted and based on serum biochemistry without imaging confirmation, all findings should be interpreted as exploratory and hypothesis-generating. Prospective studies with age-balanced sampling, pubertal staging, standardised fasting conditions and liver imaging are required to validate these observations and assess their potential clinical relevance.

## Figures and Tables

**Table 1:** Demographic, anthropometric and basic biochemical characteristics.

Variable	Obesity (n = 100)	Undernutrition (n = 90)	Normal Weight (n = 500)	*p* Value
Age (years)	11.0 [8.5–15.0]	10.0 [6.5–12.1]	7.5 [5.0–11.5]	<0.001
Sex, female, n (%)	59 (59.0)	51 (56.7)	254 (50.8)	0.236
Body weight (kg)	63.2 [43.2–77.0]	24.0 [16.0–32.2]	25.2 [17.6–40.0]	<0.001
BMI (kg/m^2^)	26.4 [23.3–30.0]	14.0 [12.9–14.8]	15.9 [14.4–17.9]	<0.001
Glucose (mg/dL)	90.4 [85.0–96.0]	89.5 [85.0–94.2]	87.0 [83.0–92.4]	<0.001
Creatinine (mg/dL)	0.5 [0.4–0.6]	0.5 [0.3–0.5]	0.4 [0.3–0.5]	<0.001
Total protein (g/dL)	7.2 [7.0–7.5]	7.1 [6.8–7.4]	7.0 [6.7–7.3]	<0.001
Albumin (g/dL)	4.7 [4.5–4.8]	4.7 [4.5–4.9]	4.6 [4.5–4.8]	0.010

Data are presented as median [IQR] or n (%), as appropriate. *p* values refer to omnibus three-group Kruskal–Wallis or chi-square comparisons. BMI, body mass index; IQR, interquartile range.

**Table 2:** Liver enzyme, lipid, micronutrient and metabolic findings.

Variable	Obesity (n = 100)	Undernutrition (n = 90)	Normal Weight (n = 500)	*p* Value
Total cholesterol (mg/dL)	156.5 [139.0–173.3]	155.2 [133.5–180.0]	152.0 [139.0–171.0]	0.478
Triglycerides (mg/dL)	87.7 [68.1–127.5]	71.0 [52.9–91.4]	69.0 [52.2–98.1]	<0.001
HDL cholesterol (mg/dL)	47.5 [42.0–56.5]	52.7 [43.3–62.0]	52.3 [45.0–60.9]	0.004
LDL cholesterol (mg/dL)	94.0 [80.7–110.0]	88.6 [78.7–110.2]	89.0 [76.3–106.7]	0.183
AST (U/L)	22.0 [19.0–26.7]	27.0 [21.0–30.0]	26.0 [21.0–31.0]	<0.001
ALT (U/L)	18.3 [14.0–25.0]	13.0 [10.9–17.0]	12.0 [10.0–16.0]	<0.001
AST/ALT ratio	1.2 [0.8–1.5]	1.9 [1.5–2.2]	2.0 [1.5–2.4]	<0.001
Iron (µg/dL)	61.3 [42.6–86.3]	73.6 [43.6–96.0]	74.7 [53.1–98.6]	0.009
TIBC (µg/dL)	359.4 [302.4–386.4]	321.5 [292.7–354.7]	322.2 [269.1–369.0]	<0.001
Vitamin B12 (pg/mL)	367.1 ± 154.6	430.1 ± 207.2	420.2 ± 151.7	0.006
25(OH)D (ng/mL)	12.8 [9.4–18.8]	18.3 [13.4–25.3]	18.4 [13.0–24.2]	<0.001
Folate (ng/mL)	8.1 [6.1–11.7]	10.7 [7.8–13.1]	9.5 [6.9–12.3]	0.017
Insulin (µIU/mL)	19.7 [15.7–27.6]	5.8 [4.5–10.2]	6.9 [4.2–13.8]	<0.001
HbA1c (%)	5.3 ± 0.3	5.2 ± 0.2	5.1 ± 0.4	0.316
Zinc (µg/dL)	95.0 [80.1–101.7]	108.0 [104.7–120.2]	82.0 [77.0–92.0]	0.016

Data are presented as median [IQR] or mean ± SD, as appropriate. Lipid and insulin values were not obtained under uniformly verified fasting conditions and should be interpreted as real-world biochemical markers. ALT, alanine aminotransferase; AST, aspartate aminotransferase; HbA1c, haemoglobin A1c; HDL, high-density lipoprotein; IQR, interquartile range; LDL, low-density lipoprotein; SD, standard deviation; TIBC, total iron-binding capacity; 25(OH)D, 25-hydroxyvitamin D.

**Table 3:** Complete blood count-derived inflammatory indices.

Variable	Obesity (n = 100)	Undernutrition (n = 90)	Normal Weight (n = 500)	*p* Value
NLR	1.5 [1.1–1.9]	1.3 [1.0–1.6]	1.3 [1.0–1.6]	<0.001
PLR	118.0 [101.5–143.4]	114.4 [95.4–141.8]	108.8 [92.2–129.5]	0.005
SII	509.6 [379.5–696.5]	440.6 [333.4–561.5]	406.3 [295.4–524.5]	<0.001
SIRI	0.8 [0.6–1.1]	0.7 [0.4–1.0]	0.5 [0.3–0.7]	<0.001

Data are presented as median [IQR]. IQR, interquartile range; NLR, neutrophil-to-lymphocyte ratio; PLR, platelet-to-lymphocyte ratio; SII, systemic immune-inflammation index; SIRI, systemic inflammation response index.

**Table 4:** Post hoc pairwise comparisons following significant Kruskal–Wallis tests.

Variable	Obesity vs. Undernutrition	Obesity vs. Normal Weight	Undernutrition vs. Normal Weight
AST	<0.001	<0.001	0.592
ALT	<0.001	<0.001	0.106
AST/ALT ratio	<0.001	<0.001	0.364
Triglycerides	<0.001	<0.001	0.543
HDL cholesterol	0.022	0.001	0.943
NLR	0.003	<0.001	0.647
PLR	0.498	0.004	0.050
SIRI	0.002	<0.001	<0.001
SII	0.024	<0.001	0.054

Post hoc pairwise comparisons were performed using the Mann–Whitney U test with Bonferroni correction. Statistical significance was defined as *p* < 0.0167. Accordingly, *p* = 0.024 and *p* = 0.050 were not considered statistically significant after Bonferroni correction.

**Table 5:** Multivariable logistic regression analysis of factors independently associated with the obesity phenotype.

Variable	B	SE	Adjusted OR	95% CI	*p* Value
AST (U/L)	0.002	0.017	1.002	0.969–1.036	0.920
AST/ALT ratio	−3.137	0.369	0.043	0.021–0.089	<0.001
HDL cholesterol (mg/dL)	−0.011	0.013	0.989	0.964–1.014	0.380
Triglycerides (mg/dL)	0.007	0.003	1.007	1.001–1.013	0.025
SIRI	1.654	0.297	5.225	2.920–9.351	<0.001
Age (years)	−0.021	0.040	0.979	0.905–1.059	0.599
Sex	−0.474	0.291	0.622	0.352–1.102	0.104

AST, aspartate aminotransferase; ALT, alanine aminotransferase; HDL, high-density lipoprotein; SIRI, systemic inflammation response index; OR, odds ratio; CI, confidence interval; SE, standard error.

**Table 6:** Age-unadjusted exploratory discriminatory performance of selected markers for the obesity phenotype.

Marker	AUC (95% CI)	Optimal Cut-Off	*p* Value	Sensitivity (%)	Specificity (%)
AST/ALT ratio	0.859 (0.823–0.895)	≤1.52	<0.001	77.0	77.6
SIRI	0.751 (0.700–0.802)	>0.70	<0.001	69.0	70.8
SII	0.644 (0.586–0.701)	>450.0	<0.001	59.0	59.8
NLR	0.634 (0.576–0.692)	>1.42	<0.001	60.0	60.0
PLR	0.583 (0.524–0.641)	>113.7	0.008	56.0	56.1

ROC analysis was performed exploratorily and without age adjustment to evaluate the ability of each index to discriminate obesity from the combined non-obesity groups (undernutrition + normal weight). Optimal cut-offs were identified by the Youden index. For the AST/ALT ratio, lower values are associated with the obesity phenotype; the optimal cut-off is therefore expressed as ≤1.52. Results are ordered by descending AUC. AUC, area under the curve; CI, confidence interval; ALT, alanine aminotransferase; AST, aspartate aminotransferase; NLR, neutrophil-to-lymphocyte ratio; PLR, platelet-to-lymphocyte ratio; ROC, receiver operating characteristic; SII, systemic immune-inflammation index; SIRI, systemic inflammation response index.

## Data Availability

The data that support the findings of this study are available from the corresponding author upon reasonable request, subject to institutional data governance requirements.
